# Lactoferrin and Human Neutrophil Protein (HNP) 1–3 Levels During the Neonatal Period in Preterm Infants

**DOI:** 10.3389/fped.2022.909176

**Published:** 2022-07-27

**Authors:** Kirstin B. Faust, Katja Moser, Maren Bartels, Ingmar Fortmann, Kathrin Hanke, Christian Wieg, Guido Stichtenoth, Wolfgang Göpel, Egbert Herting, Christoph Härtel

**Affiliations:** ^1^Department of Paediatrics, University of Lübeck, Lübeck, Germany; ^2^Department of Neonatology and Pediatric Intensive Care, Hospital Aschaffenburg-Alzenau, Aschaffenburg, Germany; ^3^Department of Pediatrics, University of Würzburg, Würzburg, Germany

**Keywords:** antimicrobial polypeptides, lactoferrin, blood levels, preterm infants, infection, inflammation, HNP 1–3

## Abstract

Antimicrobial polypeptides (APPs) are part of the innate immune system, but their specific role in the context of preterm birth is not yet understood. The aim of this investigation was to determine the systemic expression of APPs, i.e., lactoferrin (LF) and human neutrophil protein (HNP) 1–3 in preterm infants in the period of highest vulnerability for infection and to correlate these biomarkers with short-term outcome. We therefore conducted a prospective two-center study including plasma samples of 278 preterm infants and 78 corresponding mothers. APP levels were analyzed on day 1, 3, 7, and 21 of life via enzyme-linked immunosorbent assay (ELISA). The levels of LF and HNP1–3 remained stable during the first 21 days of life and were not influenced by maternal levels. Elevated APP levels were found at day 1 in infants born to mothers with amniotic infection syndrome (AIS vs. no AIS, mean ± SD in ng/ml: LF 199.8 ± 300 vs. 124.1 ± 216.8, HNP 1–3 16,819 ± 36,124 vs. 8,701 ± 11,840; *p* = 0.021, *n* = 179). We found no elevated levels of APPs before the onset of sepsis episodes or in association with other short-term outcomes that are in part mediated by inflammation such as necrotizing enterocolitis (NEC) or retinopathy of prematurity (ROP). Interestingly, infants developing bronchopulmonary dysplasia (BPD) showed higher levels of HNP1–3 on day 21 than infants without BPD (13,473 ± 16,135 vs. 8,388 ± 15,938, *n* = 111, *p* = 0.008). In infants born without amniotic infection, levels of the measured APPs correlated with gestational age and birth weight. In our longitudinal study, systemic levels of LF and HNP 1–3 were not associated with postnatal infection and adverse short-term outcomes in preterm infants.

## Introduction

Preterm infants are vulnerable for short- and long-term morbidity originating from perinatal infections and the associated inflammatory response. Their infection risk is mainly attributed to immaturity and imbalance of the neonatal immune system but also related to the need of invasive therapeutic measures. In this setting, the innate immune system as first-line defense mechanism is crucial (1).

Antimicrobial polypeptides (APPs), a large group of proteins with pleiotropic functions, are part of the innate immune system and have direct antimicrobial capacity (2, 3). APPs may also modulate immune defense pathways (4) and inflammatory processes (5). APPs are produced by epithelial cells of airway tissues and the gastrointestinal tract (6, 7). Human defensins are found at the fetomaternal interface, i.e., amnionic fluid, placenta, (8) vernix caseosa (9) and human milk (e.g., lactoferrin (LF) and LL-37) (10). Other APPs are primarily expressed by neutrophils and reach high systemic levels such as lactoferrin (LF), human neutrophil peptides (HNP) (11) or bacterial permeability increasing protein (BPI) (12).

In preterm infants, infection and inflammation may influence systemic levels of APPs (6, 7, 13–15). In line with that, LF has been proposed to have a beneficial effect by counter-regulating systemic inflammation in neonatal sepsis (16), which was not confirmed by other investigators (17). While some data exist on the influence of gestational age on the constitutive expression of LF, human beta defensin-2 and BPI (12, 18–20), expression patterns of HNP in preterm infants are unknown. As APPs are potential candidates for supplementation in vulnerable infants, we studied the systemic levels of LF and HNP in preterm infants during their highest period of vulnerability for infection, i.e., day 1–21 of life. The main objectives were to evaluate whether infants with lower plasma concentrations for LF and HNP have an increased risk for infection and whether APP levels are associated with inflammation-mediated short-term outcomes.

## Patients and Methods

### Study Population

This prospective two-center convenience sample study included a cohort of 278 preterm infants (gestational age 22 5/7–34 6/7 weeks) in the perinatal centers of the University of Luebeck Hospital (*n* = 158 infants, *n* = 78 corresponding mothers) and the hospital at Aschaffenburg-Alzenau (*n* = 120 infants).

### Ethical Approval

Ethical approval was given for all study parts by the University of Luebeck ethical committee and the Bavarian Medical Board ethical committee. Informed written consent was given by parents as legal representatives on behalf of their infants. While withdrawal of peripheral full blood counts was part of clinical routine, a maximum of additional 0.5 ml blood/kg body weight was obtained for research purposes in accordance with current guidelines of the European Medical Agency. Maternal blood samples were obtained within routine laboratory testing at delivery.

### Clinical Data and Definitions

Data were collected from clinical record files of mother and infant pairs. Gestational age was defined according to postmenstrual age (obstetrical dating). Cause of preterm delivery was defined by the attending obstetrician. Specifically, clinical chorioamnionitis (amniotic infection syndrome, AIS) was diagnosed when more than two of the following clinical signs were noted: maternal fever > 38.0°C, or fetal tachycardia > 180/min, maternal increase in white blood cell count > 10/nL or C-reactive protein levels (> 10 mg/L) without other focus of infection, painful uterus, foul-smelling amnionic fluid, preterm labor, preterm rupture of membranes or early onset sepsis in the newborn.

Small for gestational age (SGA) was defined as a birth weight less than 10th percentile according to gender-specific standards for birth weight by postmenstrual age in Germany (21).

Clinical sepsis was defined as condition with at least two signs of systemic inflammatory response (temperature > 38°C or < 36.5°C, tachycardia > 200/min, new onset or increased frequency of bradycardias or apneas, hyperglycemia > 140 mg/dL, base excess < -10 mval/L, changed skin color, increased oxygen requirements; laboratory sign: C-reactive protein > 10 mg/L, immature/neutrophil-ratio > 0.2, white blood cell count < 5/nL, platelet count < 100/nL) plus the decision of the attending neonatologist to treat with antibiotics for at least 5 days, but without growth of bacteria in blood culture (22). Blood culture proven sepsis was defined as clinical sepsis with growth of bacteria in blood culture. If coagulase-negative staphylococci (CoNS) were isolated as single pathogen in one peripheral blood culture, two clinical signs and one laboratory sign were required to fulfill the definition of blood culture confirmed sepsis. EOS was defined as sepsis occurring in the first 72 h of life. LOS was defined as sepsis occurring later than 72 h of life.

Necrotizing enterocolitis (NEC) surgery was defined according to modified Bell criteria (≥ stage 2) requiring surgery. Bronchopulmonary dysplasia (BPD) was defined as need of oxygen or respiratory support (continuous positive airway pressure (CPAP) or mechanical ventilation) at 36 weeks’ postmenstrual gestational age and retinopathy of prematurity (ROP) as ROP requiring treatment.

### Sample Collection and Analysis of Biomarkers

Plasma samples of preterm infants (*n* = 278) were collected in tubes containing 16 I.E. heparin/ml from arterial cord blood and peripheral blood on days 1, 3, 7, and 21 of life. For 78 cases, plasma samples were also collected from corresponding mothers during the first 48 h after birth within routine analysis. After centrifugation aliquots were stored at –20°C until analysis. Maximum storage time before centrifugation was 24 h. Levels of APPs were determined in plasma probes using commercial ELISA kits (Hycult Biotechnology, Netherlands), HK317 for human HNP 1–3 and HK329 for human LF according to manufacturer’s instructions. Arterial pH, glucose, total white blood cell count (WBC) and differential blood count were determined by routine hospital laboratory analysis.

### Statistical Analysis

Mann–Whitney-*U*-test and Kruskal–Wallis test were applied for statistical analysis of differences between non-paired groups. Wilcoxon test was applied for statistical analysis of differences between paired datasets. Correlations were tested by the Spearman’s rho test. The level of significance was defined as *p* < 0.05 in single comparisons and *p* < 0.01 for correlations. Statistical analysis was performed using SPSS_28.0 statistical software (SPSS Inc., Chicago, United States).

## Results

### Clinical Characteristics and Antimicrobial Polypeptide Levels

**FIGURE 1:**
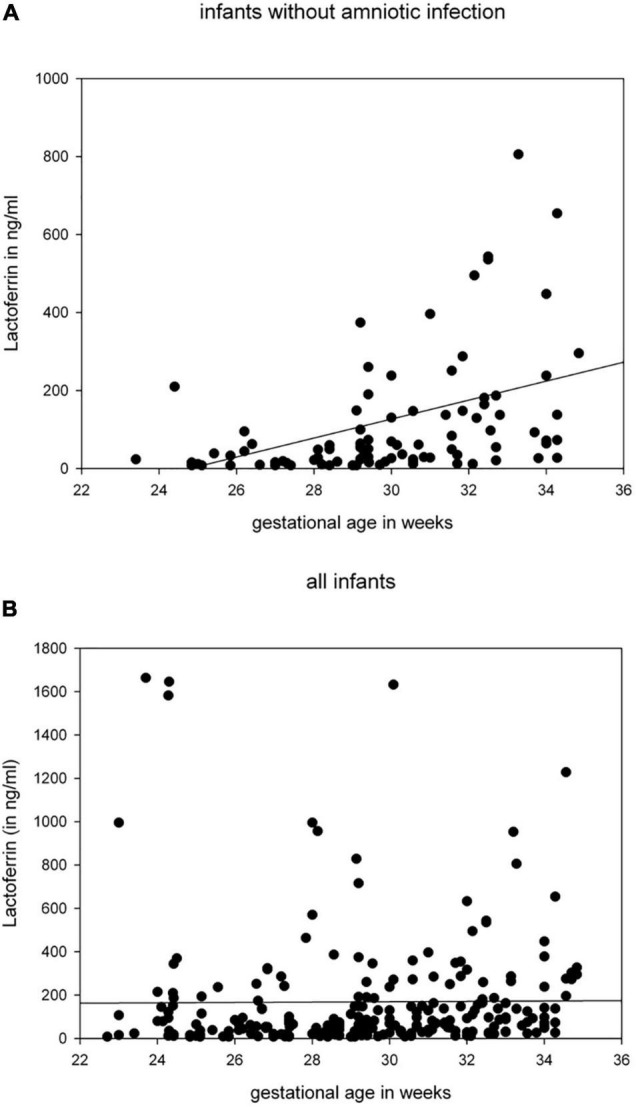
LF in ng/ml in peripheral blood of preterm infants taken at day 1 correlated with gestational age (in weeks) [**(A)** infants born without clinical signs of amnionic infection *r* = 0.491, *p* < 0.001, *n* = 96 (1 outlier not shown), **(B)** all infants].

Clinical characteristics of our study cohort are described in Table 1. We found weak correlations between gestational age or birth-weight and expression of LF levels (*r* = 0.23, *p* < 0.001, *n* = 241) in plasma of preterm infants on day 1. When we restricted our analysis to infants born without evidence of AIS (*n* = 106), we noted correlations between gestational age and levels of LF (Figure 1) as well as HNP1-3 (*r* = 0.31, *p* = 0.01, *n* = 69 (Supplementary Table 1). In line with that, LF (*r* = 0.50, *p* < 0.001, *n* = 96) and HNP1-3 levels (*r* = 0.30, *p* = 0.012, *n* = 69) were associated with birth weight in this subgroup. We found no influence of gender, multiple births or SGA on APP levels (data not shown).

**TABLE 1:** Clinical characteristics of study cohort (WBC, white blood cell; PPROM, Preterm prelabor rupture of membranes; CTG, cardiotocography; HELLP, hemolysis, elevated liver enzymes, and low platelets syndrome).

Number of infants	278
**Gestational age** (median/IQR/min-max, weeks)	29.4/12.1/22.7–34.8
**Birth weight** (median/IQR/min-max, grams)	1,325/3,070/365–3435
**Gender** (male, n/%)	156/56
**Multiples** (n/%)	109/39
**Small for gestational age** (n/%)	34/12
**WBC count/nl** (mean/median/SD)	
**d1** **d3** **d7** **d21**	13.0/10.9/8.5 10.8/8.5/7.0 12.9/11.1/7.2 14.2/12.7/7.4
**Neutrophil count/nl** (mean/median/SD)	
**d1** **d3** **d7** **d21**	4.4/2.9/4.8 6.4/5.0/5.4 6.3/4.5/6.1 5.8/4.3/5.5
**Causes of preterm delivery** (n/%)	
Preterm labor/PPROM Pathological Doppler/CTG Pre-eclampsia/HELLP Others	164/59 40/14 55/20 19/7
**Spontaneous delivery** (n/%)	46/16.2
**AIS** (n/%)	
Suspected Severe	115/41 57/21
**Early onset sepsis** (n/%)	
Clinical Blood culture proven	13/13 3/1
**Late onset sepsis** (n/%)	
Clinical Blood culture proven	19/8 30/11
**Bronchopulmonary dysplasia (BPD)** (n/%)	34/12
**Necrotizing enterocolitis (NEC)** (n/%)	10/4
**Intraventricular hemorrhage** **(IVH, any grade**) (n/%)	28/10
**Retinopathy of prematurity (ROP)** (n/%)	9/3

*One center did not perform routine blood counts on day 3.*

### Higher Antimicrobial Polypeptide Levels in Mothers as Compared to Their Infants at Birth

We detected no correlation between APP levels in infants and corresponding mothers for LF and HNP 1-3. In general, maternal APP levels were higher than cord blood or peripheral blood levels of preterm infants on day 1 [Mother/cord blood/neonatal peripheral blood d1, mean (SD) in ng/ml: LF 889 (472) / 220 (503) / 170 (272), *p* < 0.001; HNP1-3 30,750 (20,487) / 7,729 (7,611) / 13,690 (29,468), *p* < 0.001)].

### Elevated Antimicrobial Polypeptide Levels in Infants Born in the Context of Amniotic Infection Syndrome

**FIGURE 2:**
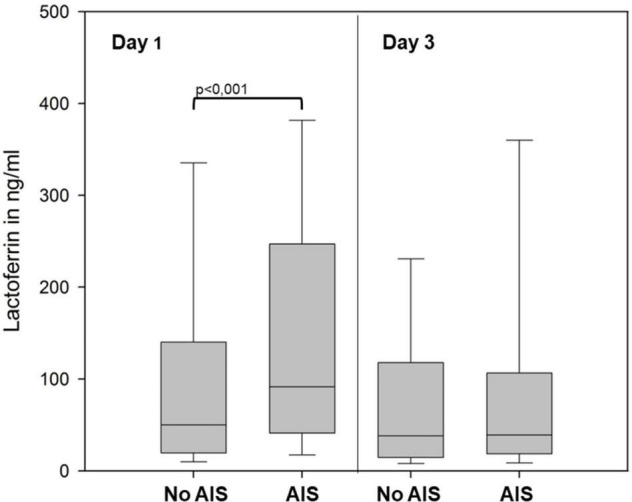
LF levels in peripheral blood of preterm infants born with (AIS, *n* = 145) or without (No AIS, *n* = 96) signs of amnionic infection at days 1 and 3. Level of significance is marked above the brackets. Data is represented as box plot (median, 25th/75th, and 10th/90th percentiles).

We found significantly higher levels of LF in infants born because of AIS on day 1 of life (Figure 2) but also for HNP1–3 (AIS vs. no AIS, mean ± SD in ng/ml: HNP 1–3 16,819 ± 36,124 vs. 8,701 ± 11,840; *p* = 0.021, *n* = 179). Mode of delivery and antenatal exposure to steroids had no impact on the levels of APPs, and no correlation was found for surrogate parameters for neonatal stress such as umbilical arterial pH or APGAR score at 5 and 10 min of life and APP levels in cord blood, day 1 or day 3 (data not shown).

### Expression of Antimicrobial Polypeptide Levels in the Period of Highest Vulnerability for Sepsis

**FIGURE 3:**
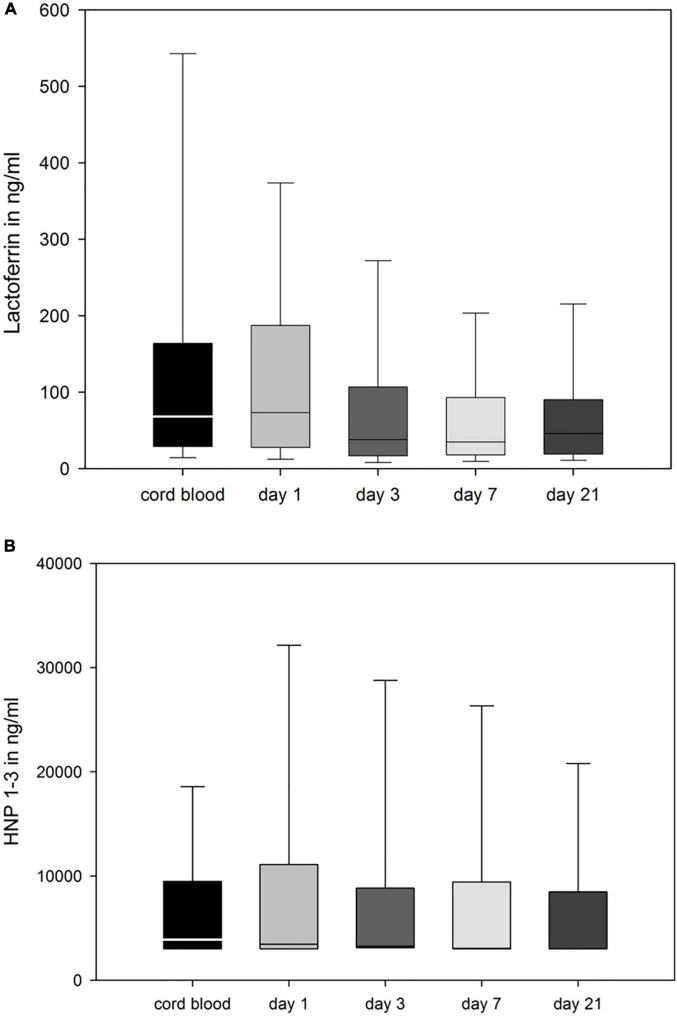
LF **(A)**, and HNP 1–3 **(B)** in ng/ml in cord blood and peripheral blood of the infant taken at days 1, 3, 7, and 21. Data is represented as box plot (median, 25th/75th, and 10th/90th percentiles).

In preterm infants, cord blood and peripheral blood levels (day 1) of APPs differed remarkably, a moderate correlation was found for LF levels only (*r* = 0.45, *p* = 0.03, *n* = 23). There was a significant correlation between levels of each APP of day 1 and day 3 (LF *r* = 0.44, *p* < 0.001, *n* = 179, HNP1–3 *r* = 0.37, *p* < 0.001, *n* = 119). The mean level of APPs remained relatively stable during the first 21 days of life (Figure 3). APP levels correlated with total white blood cell and neutrophil counts at all measured time points (Supplementary Table 2).

### Antimicrobial Polypeptide Levels Preceding Sepsis Are Not Different From Matched Samples of Unaffected Infants

No significant differences were found for APP levels preceding EOS or LOS. A trend to decreased LF levels was found for preterm infants with EOS at day 3 [mean (SD) in ng/ml, 51.9 (64.4) vs. 116.1 (208.7), *n* = 204, *p* = 0.071 and Supplementary Table 3].

Infants developing BPD showed higher levels of HNP1–3 on day 21 than infants without BPD [mean (SD) in ng/ml, 13,473 ± 16,135 vs. 8,388 ± 15,938, *n* = 111, *p* = 0.008]. We found no significant difference between APP levels in plasma of preterm infants with or without NEC or ROP (Supplementary Table 3).

## Discussion

In this prospective study we noted a correlation between amniotic infection and APP levels in preterm infants while we found no distinct pattern of APPs in association with postnatal infection. APP levels were dependent on gestational age and birth weight but also correlated with white blood cell numbers.

Amnionic infection syndrome (AIS) was associated with elevated levels of all measured APPs (LF and HNP 1–3) in peripheral blood of preterm infants at day 1. This confirms previous results showing elevated APPs in cord blood of preterm infants born due to AIS (13). Our findings may be related to transplacental passage of elevated maternal APPs. Previous studies found higher levels of APPs in plasma of mothers with amnionic infection (23, 24) as well as APP transfer at the fetomaternal interface (25). In our study, we found no correlation between APP levels of mothers and infants, while maternal levels were higher than infant levels. Additionally, APP levels in cord blood did not correlate well with levels in peripheral blood of the infants, possibly due to technical contamination with maternal blood in the cord blood samples. We therefore encourage using peripheral blood samples for future investigations.

Previous studies (12, 13, 18, 20) had shown that APP levels in cord blood may be influenced by endogenous factors such as gestational age or birth weight. This was also demonstrated in peripheral blood during the first days of life, but perinatal inflammation had a more pronounced effect in our study context. We were not able to confirm a significant decline of APP levels over time (17) in peripheral APP levels in the subgroup of infants without AIS. A subgroup of extremely preterm infants born after 23 or 24 weeks of gestation expressed very low levels of APPs if not exposed to AIS, but were able to produce APP levels similar to near-term neonates in the presence of AIS.

Within the limits of our study, we were not able to confirm our hypothesis that neonatal infection is associated with low systemic LF levels. No significant differences were found between the LF levels between infants with or without sepsis, but we noted a trend to decreased LF levels in preterm infants with EOS on day 3. Lower APP levels in neonates after birth may depend on low nutritional input, and there is evidence that oral supplementation with bovine LF may be beneficial in the prevention of NEC or LOS in very preterm infants (26). However, this is still a matter of debate and has not entered clinical routine yet. Our data do not support the use of LF as biomarker preceding sepsis.

Likewise, we found no association of HNP-levels with postnatal inflammation such as LOS, NEC or ROP. However, HNP 1–3 was elevated in infants diagnosed with another inflammation-mediated disease, BPD, comparable to serum concentrations of several cytokines (27). Interestingly, previous studies found an elevation of serum HNP in children with poor pulmonary outcome (15), assuming its function as an alarmin may be of use as a prognostic marker of the inflammation mediating the lung disease. This aspect needs to be confirmed in prospective trials in order to evaluate whether HNP adds information within the multifactorial risk profile for BPD. The role of HNPs in the immune defense is currently under discussion (2, 28), so our data may add new information to this topic.

Our study has several limitations. First, alterations of APP levels caused by pre-analytic sampling (plasma—as used in our study—vs. serum) and storage cannot be excluded. Secondly, we studied sepsis as primary endpoint, while our data cannot provide conclusive evidence on less frequent entities such as ROP, NEC, IVH or BPD. Therefore, multicenter approaches are needed to generate large scale data on the diagnostic or even prognostic value of HNP1–3. Finally, the APP monitoring was performed within routine blood sampling on day 1, 3, 7, 21 in order to minimize the burden of invasive measures for vulnerable preterm infants. Hence no samples from the exact time point of clinical suspicion of sepsis were obtained in the majority of affected infants.

## Conclusion

In conclusion, gestational age and the context of AIS correlate with APP-levels in preterm infants. Whether HNP 1–3 is a potential biomarker for diagnostic tests or therapeutic monitoring in BPD needs to be defined in further clinical studies.

## Data Availability Statement

The raw data supporting the conclusions of this article will be made available by the authors, without undue reservation.

## Ethics Statement

The studies involving human participants were reviewed and approved by the University of Lübeck Ethical Committee. Written informed consent to participate in this study was provided by the participants’ legal guardian/next of kin.

## Author Contributions

CH and KF conceptualized the study, carried out the initial data analyses, drafted the initial manuscript, and approved the final manuscript as submitted. KM and MB performed the experiments. KH, IF, CW, GS, WG, and EH supervised and coordinated the data and sample collection, supported the study design and the development of data collection instruments. All authors contributed to the manuscript, approved the final version as submitted and agreed to be accountable for all aspects of the work.

## Conflict of Interest

The authors declare that the research was conducted in the absence of any commercial or financial relationships that could be construed as a potential conflict of interest.

## Publisher’s Note

All claims expressed in this article are solely those of the authors and do not necessarily represent those of their affiliated organizations, or those of the publisher, the editors and the reviewers. Any product that may be evaluated in this article, or claim that may be made by its manufacturer, is not guaranteed or endorsed by the publisher.
