# Supplementary material for: Lactoferrin and Human Neutrophil Protein (HNP) 1–3 Levels During the Neonatal Period in Preterm Infants
Source: Front Pediatr. 2022 Jul 27;10:909176. doi: 10.3389/fped.2022.909176 (PMC9364083; doi:10.3389/fped.2022.909176)
Supplement: Supplementary file 1 [file Data_Sheet_1.pdf]

# Supplementary Tables

| | | Day 1 | Day 3 | Day 7 | Day 21 |
|---|---|---|---|---|---|
| **Lactoferrin vs. GA** | **All infants** | p<0.001<br>r=0.23<br>n=241 | p=0.001<br>r=0.228<br>n=204 | *p=0.095*<br>*r=0.123*<br>*n=185* | *p=0.873*<br>*r=0.012*<br>*n=170* |
| | **No AIS** | p<0.001<br>r=0.491<br>n=96 | p=0.002<br>r=0.336<br>n=85 | *p=0.064*<br>*r=0.213*<br>*n=76* | *p=0.977*<br>*r=0.004*<br>*n=70* |
| **HNP 1-3 vs. GA** | **All infants** | *p=0.513*<br>*r=0.049*<br>*n=179* | *p=0.93*<br>*r=0.007*<br>*n=143* | *p=0.531*<br>*r=-0.058*<br>*n=120* | *p=0.262*<br>*r=-0.106*<br>*n=113* |
| | **No AIS** | p=0.01<br>r=0.308<br>n=69 | *p=0.139*<br>*r=0.204*<br>*n=54* | *p=0.907*<br>*r=0.018*<br>*n=46* | *p=0.522*<br>*r=-0.1*<br>*n=43* |

**Suppl.1: APPs in peripheral blood of preterm infants correlated with gestational age (GA)**, at different time points (non-significant correlation in italic letters), in all infants or in a subgroup of infants born without clinical signs of amnionic inflammation (No AIS).

| | | **White blood cell count** | **Neutrophil count** |
|---|---|---|---|
| **Day 1** | **lactoferrin** | p<0,001<br>r=0,44<br>n=241 | p<0,001<br>r=0,48<br>n=241 |
| | **HNP 1-3** | p<0,001<br>r=0,51<br>n=179 | p<0,001<br>r=0,46<br>n=241 |
| **Day 3** | **lactoferrin** | p<0,001<br>r=0,37<br>n=95 | p=0,001<br>r=0,34<br>n=88 |
| | **HNP 1-3** | p<0,001<br>r=0,43<br>n=88 | p<0,001<br>r=0,37<br>n=83 |
| **Day 7** | **lactoferrin** | p=0,003<br>r=0,22<br>n=158 | p=0,003<br>r=0,22<br>n=153 |
| | **HNP 1-3** | p=0,001<br>r=0,32<br>n=96 | p<0,001<br>r=0,36<br>n=93 |
| **Day 21** | **lactoferrin** | p=0,016<br>r=0,18<br>n=143 | p=0,024<br>r=0,17<br>n=137 |
| | **HNP 1-3** | p=0,002<br>r=0,28<br>n=108 | p=0,002<br>r=0,28<br>n=103 |

**Suppl.2: APPs in peripheral blood of preterm infants correlated with white blood cell count and neutrophil count** at different time points (APPs and cell count were taken at the same day)

| | Lactoferrin (ng/ml) | | | | HNP 1-3 (ng/ml) | | | |
|---|---|---|---|---|---|---|---|---|
| | **Day 1** | **Day 3** | **Day 7** | **Day21** | **Day 1** | **Day 3** | **Day 7** | **Day21** |
| **EOS** (n=242)<br>No EOS (n=36) | 225.51<br>161.40 | 51.91<br>116.09 | 143.44<br>82.53 | 152.36<br>83.40 | 27483<br>11239 | 13739<br>9646 | 16947<br>9927 | 15976<br>8073<br>p=0.014 |
| **LOS** (n=225)<br>No LOS (n=49) | 209.03<br>160.07 | 65.92<br>114.27 | 108.30<br>87.08 | 120.45<br>82.59 | 15632<br>12060 | 13105<br>8620 | 7853<br>9869 | 14804<br>9076 |
| **BPD** (n=240)<br>No BPD (n=34) | 238.77<br>158.85 | 71.42<br>111.89 | 132.60<br>83.68 | 126.14<br>83.24 | 12663<br>12825 | 15798<br>8432 | 13578<br>8471<br>p=0.011 | 13473<br>8387<br>p=0.008 |
| **NEC** (n=263)<br>No NEC (n=10) | 256.01<br>166.30 | 120.72<br>105.62 | 122.97<br>90.49 | 80.96<br>91.88 | 37978<br>11666 | 10983<br>9442 | 12786<br>9079 | 20103<br>8956 |
| **ROP** (n= 264)<br>No ROP (n=9) | 264.93<br>200.34 | 65.84<br>88.98 | 132.32<br>90.19 | 90.01<br>88.30 | 28939<br>13944 | 12948<br>9537 | 10460<br>9783 | 17840<br>9375 |

**Suppl.3: APPs in peripheral blood of preterm infants correlated with short term clinical outcome of the infants** at different time points (mean values in ng/ml, p-value is cited only in case of significant correlation), Early-onset Sepsis (EOS, clinical or blood-culture proven), Late-onset Sepsis (LOS, clinical or blood-culture proven), Necrotizing enterocolitis (NEC, requiring surgery), Bronchopulmonary dysplasia (BPD, need of oxygen or respiratory at 36 weeks' postmenstrual gestational age) and retinopathy of prematurity (ROP, requiring treatment)
